# Spontaneous Coronary Artery Dissection, Apical Thrombus and Unknown Hyperthyroidism: A Clinical Case

**DOI:** 10.3390/jcm15134930

**Published:** 2026-06-25

**Authors:** Nicole Speziali, Giuseppe Verolino, Paolo Ghiso Basile, Roberto Adriano Latini, Davide Sala, Dario Calderone

**Affiliations:** 1 Department of Medicine and Surgery, University of Milano-Bicocca, 20126 Milan, Italy; n.speziali@campus.unimib.it; 2 Department of Invasive Cardiology, San Luca Hospital, Istituto Auxologico Italiano, IRCCS, 20122 Milan, Italy; g.verolino@auxologico.it (G.V.); r.latini@auxologico.it (R.A.L.);

**Keywords:** spontaneous coronary artery dissection, coronary intramural hematoma, intravascular ultrasound, hyperthyroidism, ventricular thrombosis

## Abstract

**Background:** Spontaneous coronary artery dissection (SCAD) is a rare non-atherosclerotic cause of acute coronary syndrome (ACS), and recent studies have shown a significant involvement in young women with few or no cardiovascular risk factors. The pathogenesis is multifactorial, possibly related to genetic causes, hormonal imbalances or peripartum. **Methods and results:** A 45-year-old woman was admitted to the emergency room for anterior myocardial infarction. Coronary angiography showed a long, diffuse tapering involving mid to distal left anterior descending (LAD) segments, with haziness in the mid-LAD. Intravascular ultrasound was used, highlighting the presence of intramural hematoma and leading to medical management. Hematological samples revealed a diagnosis of hyperthyroidism; this detection prompted an evaluation of a possible association with SCAD. Echocardiography revealed a preserved ejection fraction with akinetic apex and a sessile thrombus leading to anticoagulant with warfarin. **Discussion:** SCAD is a rare cause of ACS, difficult to recognize and whose physiopathology is not fully understood. In our case report we highlight the possible link between SCAD and hyperthyroidism. The lack of specific guidelines, the concomitant presence of hyperthyroidism and apical thrombus forced us toward a multidisciplinary management approach, with a meticulous evaluation of risks and benefits to offer the best therapeutic option.

## 1. Learning Objective

Spontaneous coronary artery dissection (SCAD) is an infrequent cause of acute coronary syndrome poorly diagnosticated and whose physiopathology is not fully understood. Hyperthyroidism is a very rare cause of SCAD, possibly leading to endothelial disfunction contributing to the chronic vascular damage underlying arterial dissection. This case report highlights the need of a comprehensive evaluation of individuals with SCAD possibly leading to multidisciplinary management in order to achieve proper management.

## 2. Introduction

Spontaneous coronary artery dissection (SCAD) is a non-iatrogenic, non-traumatic separation of the tunica intima from the adventitia of the coronary wall, not associated with atherosclerosis [1]. SCAD is the cause of acute coronary syndrome (ACS) in 0.1–4% of cases in the general population, but the prevalence reaches 22–43% if we consider women under 50 years of age [2]. The predominant mechanism of myocardial injury is extrinsic coronary artery obstruction caused by an intramural hematoma (IMH) or intimal disruption compromising the true lumen at the site of dissection [1,2]. Coronary angiography alone is not always sufficient to identify SCAD and some cases require in-depth analysis with intracoronary imaging. Indeed, intravascular ultrasound (IVUS) and optical coherence tomography (OCT) are helpful techniques to discriminate and evaluate the extension of IMH [2]. However, due to the risk of propagating the dissection, their use should be carefully considered. 

## 3. Case Presentation

**Figure 1:**
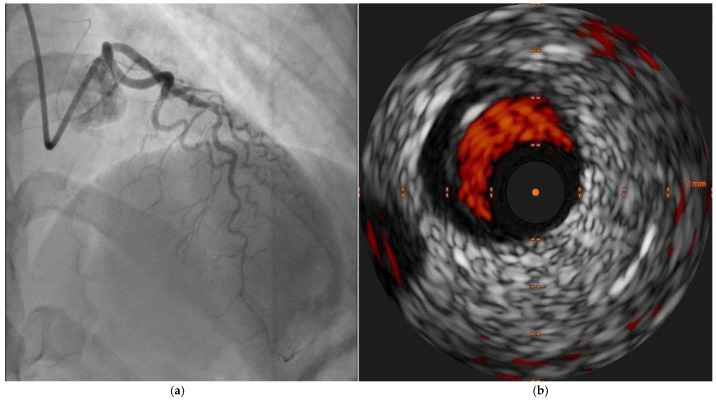
(**a**,**b**): Long tapering involving mid to distal LAD artery segments; intraprocedural IVUS with color-flow showed intramural hematoma from D1 bifurcation to apical LAD segments.

A 45-year-old woman, former smoker, with no cardiovascular history or medication on board, was admitted to the emergency room with chest pain with 12-lead electrocardiogram showing ST segment elevation in V2-V4 leads. Urgent coronary angiography showed a long tapering involving mid to distal left anterior descending (LAD) artery segments (Figure 1a), with an ambiguous haziness in the mid LAD. Because of the difficulty of angiographic differential diagnosis between type 2 SCAD and intracoronary thrombosis we decided to perform intraprocedural IVUS. IVUS highlighted the presence of a hypo-echogenic crescent-shaped area, and the color-flow system clearly discriminated the true lumen (in red, representing the blood flow, Figure 1b) from subintimal space due to the detachment of the intima from the media related to the presence of intramural hematoma, and extending from first diagonal bifurcation to apical LAD segments (Figure 1b).

**Figure 2:**
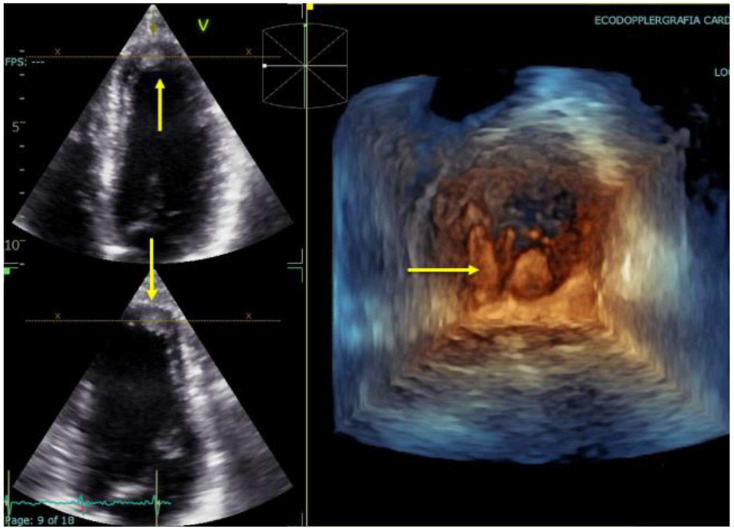
3D ultrasound echocardiography showing akinetic apex with left ventricular sessile thrombus (arrows) into the apex.

**Figure 3:**
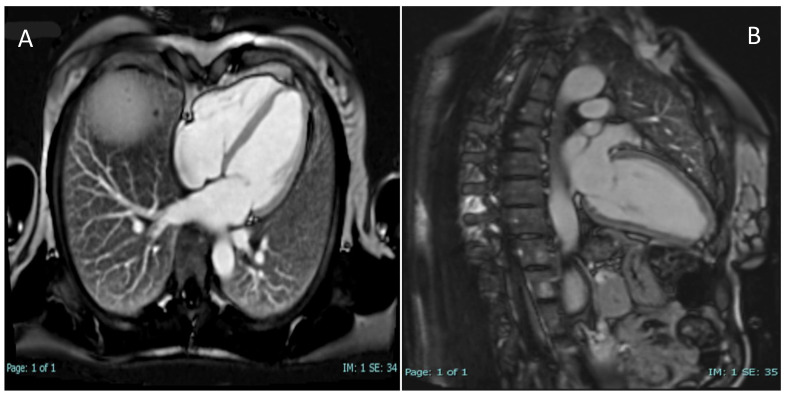
(**A**,**B**): Cardiac magnetic resonance confirming complete healing from thrombus.

The hemodynamic stability of the patient and the increased risk of coronary complications in case of percutaneous coronary intervention (PCI) in SCAD convinced us of the need for conservative medical management [1]. Meanwhile, hematological samples showed that troponin I was raised from 26 to 1227 ng/L and the thyroid stimulating hormone (TSH) was suppressed (<0.005 µU/mL). Further analyses of the thyroid profile showed fT4: 30.8 ng/dL (range 0.8–1.75 ng/dL), fT3: 13.6 pg/mL (2.3–4.2 pg/mL), anti-thyroid peroxidase antibodies 218 UI/mL (0–35 UI/mL), anti-thyroglobulin antibodies 93 UI/mL (0–40 UI/mL) and anti-TSH receptor antibodies 21.6 UI/L (0–1.5 UI/L), highlighting the autoimmune pathophysiology of autoimmune thyroid disease. Comparing these analyses with the latest available data (three months before) where thyroid hormones values were in range, a diagnosis of thyrotoxicosis (in the context of Graves–Basedow disease) was made. Methimazole was prescribed, together with cardioactive therapy with aspirin and beta blockers to reduce the risk of recurrence [2]. Fibromuscular dysplasia was excluded with abdominal and supra-aortic extracranial vessels ultrasound. No other more accurate and invasive tests were deemed necessary for the screening of fibromuscular dysplasia, since the pre-test probability was low. Transthoracic echocardiogram (Figure 2) highlighted an ejection fraction of 52% with apex akinesia. Surprisingly, an apical thrombus was observed. After a careful evaluation of the risk/benefit ratio of adding an anticoagulant (with the risk of propagating the intramural hematoma) in a patient with SCAD we decided to update the medical therapy by adding warfarin (INR range 2.0 to 3.0) to dissolve the thrombus and to reduce the risk of embolization. No additional antiplatelet therapy to aspirin was prescribed. Three months follow up showed the normalization of the electrocardiogram and hematological abnormalities. Transthoracic echocardiogram with Sonovue revealed healing from the sessile thrombus, confirmed with a cardiac magnetic resonance (CMR) scan, (Figure 3A,B) allowing discontinuation from anticoagulation with warfarin after three months from the index event. CMR showed 25% transmural late enhancement into the mid-septal wall and 75% (ischemic pattern) into the apex and the distal segments of the septum and infero-lateral wall. Global left ventricular function was preserved (63%).

## 4. Discussion

Spontaneous coronary artery dissection is a challenging cause of ACS. SCAD can be identified as a type 2 myocardial infarction, a large spectrum of non-atherosclerotic coronary mechanisms leading to a reduced myocardial oxygen supply without acute atherothrombotic plaque disruption, requiring personalized care according to the individual diagnosis and a thorough evaluation integrating clinical presentation, electrocardiographic abnormalities, biomarker kinetics, and intracoronary imaging [3].

The pathogenesis of SCAD is multifactorial and is associated with genetic causes (e.g., Marfan or Ehlers–Danlos syndromes), underlying arteriopathies (fibromuscular dysplasia) or with hormonal imbalances, and the association with female sex and pregnancy suggests that sex hormones may contribute to its development. Indeed, the role of estrogen in coronary arteries involves the activation of nitric oxide (NO) synthetase in the vascular endothelium, leading to the production of NO and subsequent vasodilation [3]. This mechanism reduces oxidative stress and promotes angiogenesis [4]. Recent evidences have suggested that SCAD often occurs during the luteal phase of the menstrual cycle, and shortly after pregnancy in postpartum females [4]. During these periods, the levels of circulating estrogen and progesterone typically decline, implicating ‘estrogen withdrawal’ or relative changes in the level of circulating estrogen and progesterone in females as a potential hormone hypothesis for SCAD [4].

Similarly, thyroid defects became a new field for investigation in SCAD. In particular, in light of the limited evidence currently available, a potential association between these two conditions can only be postulated and the literature data reviewed herein serve solely to highlight possible underlying pathophysiological mechanisms. It is known that thyroid dysfunction is associated with increased oxidative stress: hyperthyroidism causes an elevation of reactive oxygen species (ROS) and hypothyroidism is associated with a lower availability of antioxidants. The relationship with SCAD and hypothyroidism is more represented in the literature and the hypothesis relates SCAD to coronary endothelial dysfunction, but the specific mechanisms are not entirely clear. Isoforms of the thyroid hormone receptor have been identified in human aortic vascular smooth muscle cells, suggesting that the thyroid hormone may act directly on the vascular bed and influence vasomotion [5]. Previous studies have also shown that hypothyroidism is associated with a low-grade systemic inflammation, which may act as a potential trigger [5]. The possible relation between SCAD and hyperthyroidism is even less clear. Only limited data are available on this topic: a case report [6] and a case–control study [7] of patients with spontaneous cervical artery dissection and thyroid autoimmunity. In the case report [6] the authors considered thyroid storm as a possible trigger of SCAD, hypothesizing a link between the high levels of circulating thyroid hormones and SCAD susceptibility. In the case–control study by Pezzini et al. [7], the authors supposed that immunologic mechanisms may contribute to the chronic vascular damage underlying arterial dissection, suggesting autoimmunity as a biologic determinant of SCAD [7]. Indeed, even if autoimmune thyroiditis is an “organ-specific” disease, it is associated with a systemic immune dysregulation, as suggested by the presence of a certain degree of peripheral immune deficiency in the blood of patients with this disease [7]. Therefore, spontaneous artery dissection might be one phenotypic expression of a generalized activation of immunity. It is reasonable to hypothesize that the activation of an immune-mediated process may be involved in the pathogenesis of SCAD, probably by determining an underlying susceptibility state to disease occurrence. Other studies have revealed how autoimmune processes in the thyroid gland may be responsible for systemic low-grade inflammation, which plays a relevant role in the atherosclerotic process and in the stiffening of arteries, highlighting the role of thyroid disorders as potential cardiovascular risk factors [8]. Indeed, the increase in cytokine levels (such as Interleukin 1 and 6, tumor necrosis factor-β) and reactive oxygen species plays a role in the degradation of elastin, migration of smooth muscle cells and increase in collagen in the arterial wall, leading to a dysregulation of smooth muscle cell tone and endothelial dysfunction [8].

Notably, regardless of the pathogenetic cause and clinical presentation, the angiographic features of SCAD are quite similar and standardized. In 2013 Saw proposed an angiographic classification of SCAD which nowadays is widely accepted and used [9]. Individuals presenting with hemodynamic instability, recurrent ischemia, ventricular arrhythmias and/or the involvement of the left main coronary artery can benefit from revascularization [1]. However, when diagnostic uncertainty exists or to guide coronary intervention when required, careful intracoronary imaging use appears reasonable [2]. Indeed, during the last decade, intracoronary imaging represented a decisive turning point for diagnosing SCAD, especially in doubtful cases. Both IVUS and OCT can be used to visualize the vessel wall, and to identify the intimal rupture, the false lumen or the intramural hematoma. Although both methods are useful, IVUS is probably preferable to OCT runs, since it does not require pressurized contrast injection and secondly because of the superior depth penetration which enables the complete visualization of the vessel wall to the external elastic lamina [2]. Recent European Society of Cardiology guidelines for the management of ACS suggest considering the use of intravascular imaging in SCAD or to guide PCI (IIa, A) or for additional help in the case of ambiguous culprit lesions (IIb, C) [10]. However, current recommendations agree on conservative treatment in most situations [1,2], as enhanced in the large, multicentric, Canadian SCAD study, supporting conservative therapy as the first line when possible [11]. In particular, beta blockers seem to decrease the risk of recurrency, possibly by reducing coronary arterial shear stress and reversing catecholamine-mediated cardiac dysfunction [2]. The role of antiplatelets for SCAD is still not clear, even if the efficacy of secondary prevention with aspirin in ACS and its low side effect profile make it reasonable to use [2].

The management of our patient was also complicated by the presence of left endoventricular thrombosis, leading to the addition of oral anticoagulant despite the risk associated with propagating the IMH and a higher risk of bleeding. However, a multidisciplinary management approach involving cardiologists, radiologists, endocrinologists and hematologists led to a comprehensive evaluation of the risk–benefit ratio of each therapy prescribed and to complete healing from a potentially fatal disease.

## 5. Conclusions

SCAD is a challenging cause of ACS often related to difficult diagnosis and management. In our case, SCAD was associated with hyperthyroidism and the detection of endoventricular thrombosis, which required further changes in the medical therapy. Because of the diverse implications and precipitating factors related to SCAD, a thorough, tailored management of this condition is required to achieve the resolution of symptoms and clinical stability.

## Data Availability

The authors confirm that the data supporting the findings of this study are available within the article. Raw data of this study are available from the corresponding author, upon reasonable request.
